# Influence of a Non-Ionic Surfactant in the Microstructure and Rheology of a Pickering Emulsion Stabilized by Cellulose Nanofibrils

**DOI:** 10.3390/polym13213625

**Published:** 2021-10-21

**Authors:** Jorge Velásquez-Cock, Angélica María Serpa, Catalina Gómez-Hoyos, Piedad Gañán, Manuel Romero-Sáez, Lina María Vélez, Natalia Correa-Hincapié, Robin Zuluaga

**Affiliations:** 1 Programa de Ingeniería en Nanotecnología, Universidad Pontificia Bolivariana, Medellín 050031, Colombia; catalina.gomezh@upb.edu.co; 2 Facultad de Ingeniería Agroindustrial, Universidad Pontificia Bolivariana, Medellín 050031, Colombia; angelicaserpa31@gmail.com (A.M.S.); lina.velez@upb.edu.co (L.M.V.); robin.zuluaga@upb.edu.co (R.Z.); 3 Facultad de Ingeniería Química, Universidad Pontificia Bolivariana, Medellín 050031, Colombia; piedad.ganan@upb.edu.co; 4 Grupo Calidad, Metrología y Producción, Instituto Tecnológico Metropolitano, Medellín 050034, Colombia; manuelromero@itm.edu.co (M.R.-S.); nataliacorrea@itm.edu.co (N.C.-H.)

**Keywords:** Pickering emulsions, non-ionic surfactant, nanocellulose, coconut oil, colloids

## Abstract

Emulsion stabilization is a broad and relevant field with applications in oil, polymer and food industries. In recent years, the use of solid particles to stabilize emulsions or Pickering emulsions have been studied for their kinetic and physical properties. Nanomaterials derived from natural sources are an interesting alternative for this application. Cellulose nanofibrils (CNFs) have been widely explored as a Pickering emulsifier with potential food applications, however, in some cases the presence of surfactants is unavoidable, and the literature is devoid of an evaluation of the effect of a non-ionic food-grade surfactant, such as polysorbate 80, in the stabilization of a vegetable oil by CNFs. To better assess the possible interactions between CNFs and this surfactant emulsions containing coconut oil, an emerging and broadly used oil, were processed with and without polysorbate 80 and evaluated in their qualitative stability, morphological and physical properties. Fluorescence microscopy, dynamic light scattering and rheology were used for this assessment. Results indicate in absence of the surfactant, emulsion stability increased at higher CNFs content, creaming was observed at 0.15 and 0.3 wt.% of CNFs, while it was not evidenced when 0.7 wt.% was used. After the addition of surfactant, the droplets are covered by the surfactant, resulting in particles with a smaller diameter, entrapped in the cellulosic structure. Rheology indicates a lower network stiffness after adding polysorbate 80.

## 1. Introduction

The mixture of two immiscible liquids, such as oil and water in a fine dispersion of droplets surrounded by a continuous medium, form a thermodynamically unstable colloid, known as an emulsion. The stabilization of these dispersions has implications in the pharmaceutical, cosmetic, food and petrochemical industries, among other applications [1,2]. The formation of emulsions has been accomplished by low-molecular weight surfactants, which adsorb in the oil-water interphase, reducing their surface tension and stabilizing the colloid [2,3]. However, it is possible to replace them with amphiphilic solid particles, relying on the coverage of the discrete phase by the solid, forming Pickering emulsions [4,5]. The stability of this type of emulsions depends on the affinity between the particle and interphase, as well as the diameter of the solid. Smaller particles tend to have a better packaging around the droplet and higher energies of desorption [4,6].

The development of nanotechnology and preparation of smaller particles have renewed the interest in this type of emulsions, as it is possible to produce particles that, in theory, will not desorb at room temperature, creating a mechanical barrier to coalescence, resulting in highly stable colloids [7].

Different compounds have been studied to develop Pickering emulsions, such as silica nanoparticles, bentonite, calcium carbonate, polystyrene, to name the most common materials [6,7,8]. However, the expanding applications of these emulsions, coupled with their use in pharmaceutical and food products [5,9,10] have shifted research to explore organic and label friendly compounds such as starch, proteins [4], lignin [11], cellulose nanocrystals (CNCs) [12] and nanofibrils (CNFs) [13,14,15] or a combination of CNCs and CNFs [16], among other compounds. The use of cellulosic products is interesting, as it is derived from natural sources and is widely available, representing nearly 1.5 × 10^12^ t of the total annual biomass production [17].

Cellulose is a biopolymer composed of repeating units of glucose linked by β 1 → 4 glycosidic bonds. During their synthesis, the cellulosic chains interact by hydrogen bonding and hydrophobic stacking, forming crystalline and amorphous regions [12,17]. Cellulose can be further processed by chemical or mechanical treatments to obtain CNCs or CNFs, respectively. CNFs are composed of free fibrils and fibril bundles with diameters below 100 nm and lengths of a few micrometers [18,19]. The presence of a flexible fibril and high aspect ratios can be advantageous to manufacture Pickering emulsions, since it decreases the percolation threshold and the extension of the coverage density by random jammed packing [12]. Anisotropic particles have been associated with more stable emulsions even at concentrations 20 times lower than spherical particles [4,5,20]. Furthermore, its biodegradability, its role as a non-soluble dietary fiber and recent developments in the food sector make this compound a relevant alternative to stabilize food-related emulsions [21].

The use of cellulose nanomaterials as a stabilizer in Pickering emulsions has been widely studied [11,12,13,21], with a clear indication that cellulose concentration as well as morphological and surface properties could be modified to stabilize different emulsions, usually prepared with model non-polar phases such as dodecane, toluene, tetradecane, among others [7,12,22,23]. These solvents help to understand the underlying physics in the stabilization of Pickering emulsions by cellulose nanomaterials, CNCs [12,22] or CNFs [22,24]. Nevertheless, the complexity of commercial oils could pose a new set of complications in the manufacture of Pickering emulsions, possibly limiting some of the promising uses of cellulose nanomaterials, for instance, in the food industry [21]. This concern has been noticed by the scientific community, and a growing body of research has been dedicated to study the addition of CNCs or CNFs to stabilize oil in water (O/W) emulsions with sunflower [16], soybean [15] or palm oil [10], leading to possible developments in the food industry.

To the authors knowledge, the existing literature has a very limited number of studies relating to the use of coconut oil in Pickering emulsions stabilized by cellulose nanomaterials [25]. The evaluation of this oil is relevant for future developments, since its production reached nearly 55 × 10^6^ t in the year 2019, and it is expected to develop a USD 4.7 billion market by 2024 [26]. It is increasingly used in different food formulations due to its high content of saturated fats, resulting in a higher resistance to the oxidative damage [25] and its characteristic flavor. Additionally, the increasing demand of this oil in the food industry underscores the need to assess the use of other novel ingredients, for instance, cellulose nanomaterials, to stabilize emulsions containing coconut oil for food applications.

The performance of cellulose nanomaterials as emulsifiers can be modified using surfactants, for instance, cationic surfactants cetyl triammonium bromide (CTAB) and dodecyldimehtylammonium bromide (DMAB) were added to dodecane in water emulsions. CNCs obtained by sulfuric acid hydrolysis were used as solid particles [7], and it was observed that electrostatic interactions had an important role, as surfactants were attracted to the cellulosic surface and could be fine-tuned to affect the type of emulsion formed [7]. Nanocellulose has also been reported to interact with non-ionic surfactants through their hydrophilic head, resulting in the attraction of the polymer to the surfactant, as observed in Triton™ X-100 [27], and has been exploited to form cellulosic foams comparable to polyurethane, in the presence of pluronic P123 [23]. This interaction could also be used to develop novel types of food. In some cases, the use of a surfactant is not optional, for instance, in ice cream the surfactant helps to create a characteristic colloidal structure [28], making the interactions between CNFs and surfactants commonly used in foods a relevant topic. Even though CNFs have been extensively studied with non-edible surfactants, such as sodium dodecyl sulfate [23], pluronic 123 [29] and triton-X100 [27], the literature is devoid of studies on the presence of cellulose nanomaterials with food-grade, non-ionic surfactants, such as polysorbate 80 (Tween™ 80), its molecular structure is in Figure S1 and its hydrophilic groups hydrate in the presence of water, allowing the emulsification of fat compounds. It is widely used in foodstuff [30]. 

Because CNCs and CNFs have different stabilization mechanisms [16], and considering the ample use of unmodified CNFs in food applications [21], the present work assessed the effect of the food-grade surfactant polysorbate 80 in the behavior of Pickering emulsions with CNFs and coconut oil. Emulsions with and without surfactant were manufactured and evaluated in their stability and morphological features by fluorescence microscopy and laser diffraction. Rheological behavior of the resulting suspension was measured as well.

## 2. Materials and Methods

### 2.1. Preparation of Cellulose Nanofibrils

Cellulose nanofibrils were isolated according to the protocol defined in our previous work [19,31]. Briefly, ground banana rachis, harvested in a banana plantation in Urabá, Antioquia, Colombia, was subjected to an alkaline treatment with a 5 wt.% solution of potassium hydroxide, for 14 h; followed by a delignification at 70 °C for 1 h with sodium chlorite at a pH of 4. The insoluble material was processed by a second alkaline treatment for 14 h and an acid demineralization treatment with hydrochloric acid at 80 °C for 2 h. The reagents used during this processing are of commercial quality.

The resultant cellulosic product was thoroughly washed and redispersed in distilled water (water type III), and passed through a grinding equipment 30 times, as described in Velásquez-Cock et al. [19]. The obtained product was sterilized and stored at 4 °C before its use. A portion of the resultant CNFs was processed and analyzed in their morphological features by an atomic force microscope (Nanosurf, Liestal, Switzerland), images were taken in dynamic mode in air, with aluminum coated silicon tips (PPP-NCSTR-10, Nanosensors, Neuchatel, Switzerland), according to the protocol described by Gómez Hoyos et al. [32]).

The charge of the resultant CNFs was 72 ± 6 μmol·g^−1^, determined by SCAN-CM 65:02 [33]. A portion of CNFs was diluted in distilled water until a concentration of 0.1 wt.% was achieved, and the resultant suspension was vacuum filtered and dried at 40 °C for 96 h.

To assess the affinity of CNFs towards the continuous and discreet phases, contact angle measurements were performed on the oven-dried films. The films were cut in 1 cm-wide strips and were adhered to a flat surface. A drop of buffer or coconut oil was deposited over the surface. Results were recorded after 30 s of contact between the drop and the surface. The resultant contact angle was measured using a goniometer (OCA15, Dataphysics, Filderstadt, Germany). Measurements were performed in triplicate.

### 2.2. Processing of Surfactant-Free Emulsions

**Figure 1:**
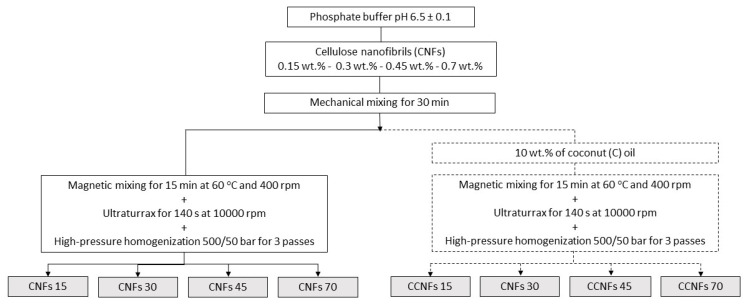
Scheme of the processing of suspensions of CNFs without oil and of emulsions of CNFs with coconut oil. CNFs contents of 0.15, 0.3, 0.45 and 0.7 wt.% were evaluated. Oil-free suspensions are CNFs15, CNFs30, CNFs45 or CNFs70, respectively. Emulsions with CNFs and coconut oil are CCNFs15, CCNFs30, CCNFs45 and CCNFs70.

Emulsions were performed based on the method developed by Winuprasith et al. [15]. CNFs were dispersed in an aqueous phosphate buffer (pH = 6.5 ± 0.1), using a mechanical mixer for 30 min; concentrations of 0.15, 0.3, 0.45 and 0.7 wt.% of the cellulosic material were used. A food grade commercial coconut oil (C) from a local provider (Bio oil, Medellín, Colombia), was added to the suspensions until a 10 wt.% content was achieved [15]; the mixture was stirred at 400 rpm and 60 °C for 15 min. Then, it was processed through a high-speed rotor-stator system (Ultraturrax T50, IKA, Cologne, Germany), impeller model S50N-G45M) at 10,000 rpm for 140 s, stopping to avoid thermal damage to the samples due to high shear stresses. Finally, samples were passed three times through a high-pressure homogenizer (Panda 2K, Niro Soavi, Parma, Italy) using both disintegration stages at 500/50 bar, [15]. An S-type impact valve and a flat head were used in the first stage, while an alumina sphere was used in the second stage. The processing flowchart is depicted in Figure 1. Samples with coconut oil and CNFs were named as CCNFs15, CCNFs30, CCNFs45 and CCNFs70, depending on the amount of nanocellulose used in each sample.

The lipid profile of the coconut oil used is recorded on Table S1. It shows a content of saturated oils above 90 g/100 g oil, which is coherent with this type of product [34].

A portion of each emulsion was stored in a cylindrical glass container (Diameter: 27 mm, Height: 111 mm), and photographs of these samples were taken at 1, 24 and 120 h to assess suspension stability. Another portion of the specimens was stored in a closed container for future analysis by microscopy and rheological equipment.

The mechanical processing of the emulsions might disrupt the fibrillar structure of the CNFs [19]. To account for this effect, oil-free suspensions were processed following the same procedure and named as CNFs15, CNFs30, CNFs45 and CNFs70. These samples were evaluated in their rheological properties and particle size distribution.

### 2.3. Rheological Analysis

Rheological behavior of the suspensions without oil was performed according to the method proposed by Saelices and Capron. [22]. The suspensions were analyzed in a stress-controlled rheometer (DHR2, TA Instruments, New Castle, DE, USA), using a texturized 40 mm plate-plate geometry, and a fixed gap between the plates of 0.5 mm. All measurements were performed at 20 °C, following an equilibration time of 10 min [35], a solvent trap was used to avoid solvent loss during the measurements. Amplitude viscoelastic measurements were performed using a frequency of 6.28 rad/s and an amplitude sweep between 0.01 and 100%. A frequency sweep was performed in the lineal viscoelastic region. Strain sweeps measurement were performed from 0.07 to 100% and from 100 to 0.07%, a fixed frequency of 6.28 rad s^−1^ was chosen. Values of the energy storage (G′) and loss moduli (G″) were recorded. Samples were analyzed in triplicate.

### 2.4. Fluorescence Microscopy Analysis

To observe the morphology of CNFs and fat globules structures, a direct observation was performed using a fluorescence microscope (Zeiss Axio Observer, Zeiss, Jena, Germany), at magnifications of 100× and 400×. Samples were prepared by staining 1 mL of fresh nanocellulose and oil suspension with 8 µL of Nile Red to dye fat structures (excitation spectrum 488 nm and emission spectrum 539 nm) [15,16]. A 6 µL drop of this suspension was mixed with 6 µL of Calcofluor white to selectively coat CNFs (excitation spectrum 365 nm and emission spectrum 435 nm). Samples were covered using a glass coverslip (18 × 18 × 2 mm^3^) and the borders were protected with nail polish to fix the borders of the coverslip and prevent evaporation [16].

### 2.5. Processing of Emulsions with CNFs and Surfactants

To assess the coverage of polysorbate 80 (P) around the oil surface before formulating the emulsion, the interfacial tension between coconut oil and a buffer solution containing the surfactant was measured using a goniometer (OCA 15, Dataphysics, Filderstadt, Germany). A drop of the aqueous phase was submerged in a glass cuvette containing oil at 50 °C. The system remained for up to 30 min to allow the diffusion of surfactant towards the interphase [8]. Pictures were analyzed using the Pendant drop plugin of image J [36]. Polysorbate 80 concentration was varied between 0.1, 0.2, 0.4, 0.6 and 1 wt.% to identify the effect of the surfactant content on the interfacial tension and the start of a plateau. The amount of polysorbate added to the suspensions is defined as the minimum quantity required to reach the surface tension plateau.

Emulsions were obtained by adding the defined amount of polysorbate 80 to suspensions containing CNFs, they are subsequently mixed with coconut oil and processed according to the procedure described in Section 2.2. Coconut oil, polysorbate 80 and different amounts of CNFs are processed as described in Section 2.2 and using a similar flow diagram. Samples were named CPCNFs15 and CPCNFs70, depending on the amount of CNFs used.

Rheological and morphological analysis of the obtained emulsions was assessed as described in Section 2.3 and Section 2.4. Particle size was evaluated by laser diffraction (Mastersizer 3000, Malvern Panalytical, Malvern, UK), suspensions were stored for 1 week before measurements. Refractive indexes of the dispersant media and for the disperse phase were 1.33 and 1.46, respectively.

## 3. Results and Discussion

### 3.1. Processing of Surfactant-Free Emulsions

**Figure 2:**
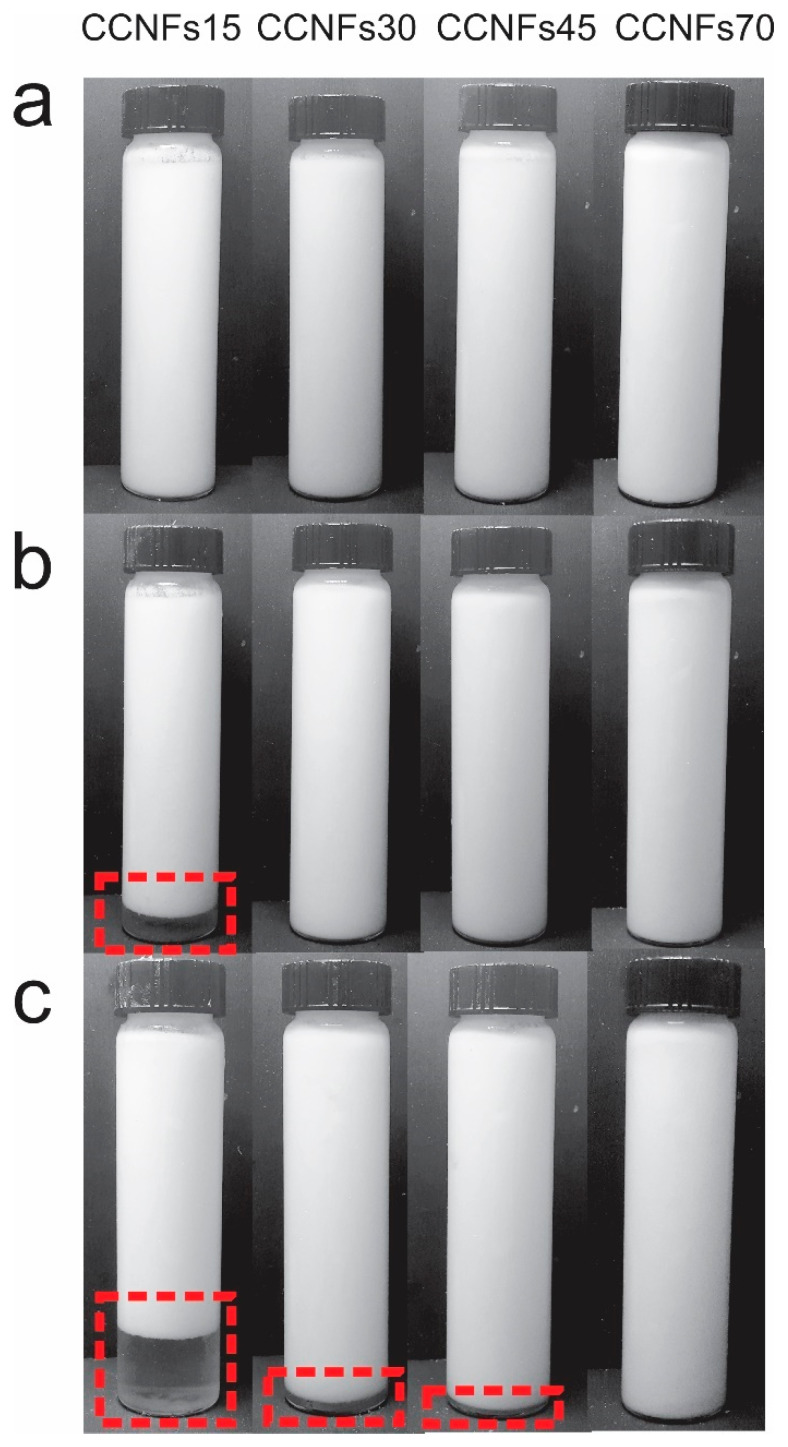
Emulsions manufactured with coconut oil, after 0 h (**a**), 1 h (**b**) and 5 days (**c**) of static storage. Dashed-line rectangle encloses the creaming of the suspension.

The obtained CNFs consists of an entangled network of fibrils and fibril bundles, Figure S2a, where most of their diameter lies between 5 and 100 nm, with a small amount of subfibrillated material above this range, Figure S2b, which is coherent with previous reports [19]. After mixing the CNFs with the other ingredients at 60 °C and obtaining the emulsions, samples were photographed at 0, 1 and 120 h after homogenization (Figure 2). As observed, one phase was distinguished immediately after their processing, and was maintained at different times, depending on the content of CNFs used.

Low amounts of CNFs resulted in a reduced stability, as evidenced in Figure 2. After one hour, CCNFs15 formed two phases, enclosed by a dashed-line rectangle. A clear phase at the bottom of the flask and a white suspension at the top. This is indicative of a creaming process [15]; where droplets flocculate, forming clusters without losing their identity to form larger droplets or coalescence. Since the clusters have a lower density than the buffer, they float towards the top of the emulsion [16]. This behavior was also evidenced in CCNFs30, while it was absent in CNFs concentrations above 0.45 wt.%. The effect of the concentration is attributed to two simultaneous phenomena, the presence of a higher amount of material to cover the oil surface [5,12]; and an increment in the viscosity of the surrounding medium, resulting in a slower flocculation or coalescence of oil droplets [16,37]. None of the emulsions showed the formation of a separate oil phase, indicating that there was no extensive coalescence and emulsion breakage during the period evaluated.

In fact, rheological measurements of a blank composed of different CNFs concentrations without oil and after the homogenization (Figure S3 of the Supplementary Information) shows that independently of the amount of nanofibrils used, it exhibits a gel-like behavior, with a storage (G′, filled symbols in Figure S3) modulus above the loss modulus (G″, blank symbols in Figure S3) up to an oscillatory deformation of 100%. This structure is associated to the formation of an interconnected network of entangled fibrils [38,39], as observed by AFM, Figure S2a. It has a lineal viscoelastic zone, up to 1%, which is not affected by cellulose concentration. After this amplitude was reached, the material exhibited a typical non-lineal behavior, leading to the break of the ordered structure the moduli crossover was not observed in the amplitude range evaluated.

Nanofibrils content influenced both G′ and G″ of the obtained emulsions since they increased along with cellulose concentration. The most important differences were between CNFs15 and CNFs30, with increments in G′ up to 395% at an amplitude of 100%, and between CNFs45 and CNFs70, where G′ was enhanced up to 39.19% at the same amplitude. A similar behavior was observed by Pääkko et al. [39] in enzyme-pretreated microfibrillated cellulose, reporting a plateau in G′ near 0.5 wt.%, followed by a steady increase up to 5.9 wt.%. The increase in both moduli implies the formation of a stronger gel structure [15], limiting the flow of oil droplets through the continuous phase [40]. The use of bacterial cellulose has shown stable suspensions up to a year at concentrations of 1.23 wt.%, [41], while the oscillatory measurements indicate that the lineal viscoelastic zone goes to a deformation of 0.1%. Network strength and stability depends on the source and the mechanical treatment of the sample, explaining the differences observed [38].

These differences in the rheological behavior of the entangled network might influence the microstructural features of the obtained CNFs, which in turn can help to explain differences in the stability of the product. Thus, to better assess the structure of the emulsions manufactured, they were analyzed by fluorescence microscopy.

**Figure 3:**
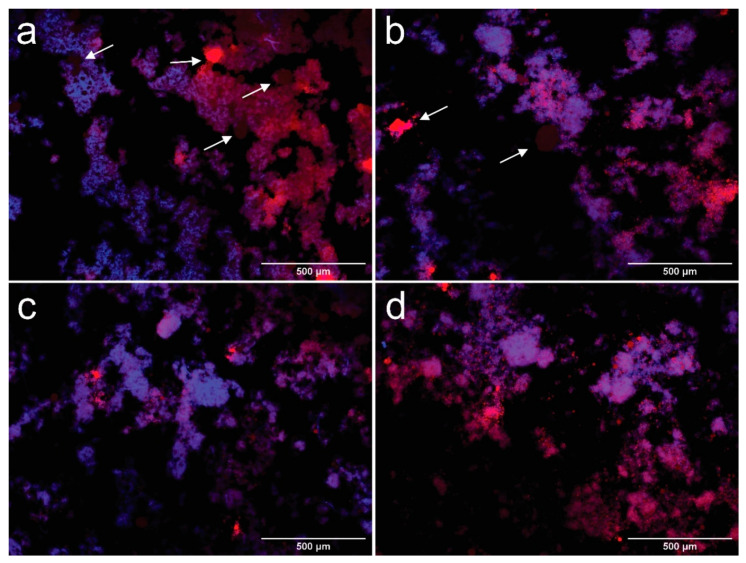
Fluorescence microscopy images taken at 100× of samples CCNFs15 (**a**), CCNFs30 (**b**), CCNFs45 (**c**), CCNFs70 (**d**) after 1 day of their manufacture. CNFs are colored in blue and oil droplets are colored in red. Coalesced oil structures are pointed by arrows in the images.

Droplet morphology was observed by fluorescence microscopy one day after processing the emulsion, as described by Bai et al. [16]. Suspensions were previously dyed with Nile red and calcofluor white to identify their different components; a drop was placed over a microscope slide and a glass coverslip was used to cover the sample before their observation at 100×. Results are recorded in Figure 3a–d.

In Figure 3a–d, it is observed that oil in water (O/W) emulsions were produced with oil droplets (red) surrounded by an aqueous medium with dispersed CNFs (blue). The contact angle between CNFs and the phosphate buffer was 62.93 ± 2.66°, which is below 90° and indicates that the formation of a O/W emulsion [42]. The processed suspensions show a heterogeneous structure, where droplets can be divided into a fine dispersion of oil droplets embedded in a cellulosic network, and a rough distribution of coalesced oil droplets, marked by arrows in Figure 2a,b. Fractions of the coalesced product are trapped inside the cellulosic network, while other zones are not covered by CNFs. The extent of the apparent coverage and the size of the flocs formed by the cellulosic network depend on the amount of cellulose used. 

Coalesced structures are formed when smaller droplets collide, forming a larger droplet, due to their hydrophobic behavior [43], they are more evident in CCNFs15 (arrows in Figure 2a), and coexist with oil droplets entrapped in a cellulosic structure. Coalescence is reduced in CCNFs30 (Figure 2b), while CCNFs45 and CCNFs70 (Figure 2c,d) do not show an extensive droplet destabilization, as most of the oil is entrapped in the flocs formed by the entangled 3D cellulosic network. The contact angle between coconut oil and CNFs was 35.28 ± 3.7°, indicating an affinity between the cellulosic product and oil, leading to its entrapment.

Microscopy observations are corroborated by previous studies on Pickering emulsions, where higher amounts of CNFs provide more coverage to the oil droplets formed, restricting their coalescence [12,15,22]. Tang et al. [42] indicated that increasing the content of CNFs contributed to the formation of a denser barrier that prevented further coalescence of toluene or hexadecane droplets. Alternatively, cellulose has been reported as an efficient depletion inducer [11]. Depletion is a colloidal phenomenon, where two particles surrounded by non-adsorbing polymers are in close proximity, resulting in a depletion zone between the particles and their consequent attraction, due to the reduced osmotic pressure among them [44]. In case of CNFs, lower concentrations of polymer might destabilize the emulsion, leading to droplet flocculation and, possibly, their coalescence. However, at higher contents it stabilizes the suspension by a depletion stabilization mechanism [16], as larger amounts of cellulose increase the energetic barrier for the droplets to flocculate [44].

In the present work, the concentration of CNFs played a crucial role in the stabilization of O/W emulsion, and the highest phase separation was observed at samples with 0.15 wt.% of CNFs, Figure 2. Creaming was not evidenced in the sample containing 0.7 wt.% of CNFs, as indicated by the absence of a phase separation in Figure 2. In this emulsion the increase of CNFs in the surrounding medium can lead to a depletion stabilization [16], while the increase in medium viscosity restricts the movement of oil droplets, enhancing its stability. The behavior of CNFs might be affected by the addition of a surfactant, which could compromise its use in food formulations, where the use of a small molecule surfactant is required, such as in ice creams [45]. Therefore, to evaluate the influence of a food-grade, non-ionic surfactant in the stability of the CNFs-coconut oil emulsions, polysorbate 80 was added to the lowest and the highest concentration of CNFs evaluated, measuring the differences between the 0.15 and 0.7 wt.% emulsions.

### 3.2. Processing of CNFs-Surfactant Emulsions

Before preparing the emulsions with polysorbate 80, the amount surfactant used was defined by the surfactant concentration required to cover the oil structure, as determined by the process indicated by Pichot et al. [8] and analyzed by the pendent drop method [36]. Results are recorded in the Table S2. As can be observed, surface tension does not exhibit a statistically significant difference beyond 0.6 wt.%, therefore, this concentration was chosen for the subsequent emulsions.

**Figure 4:**
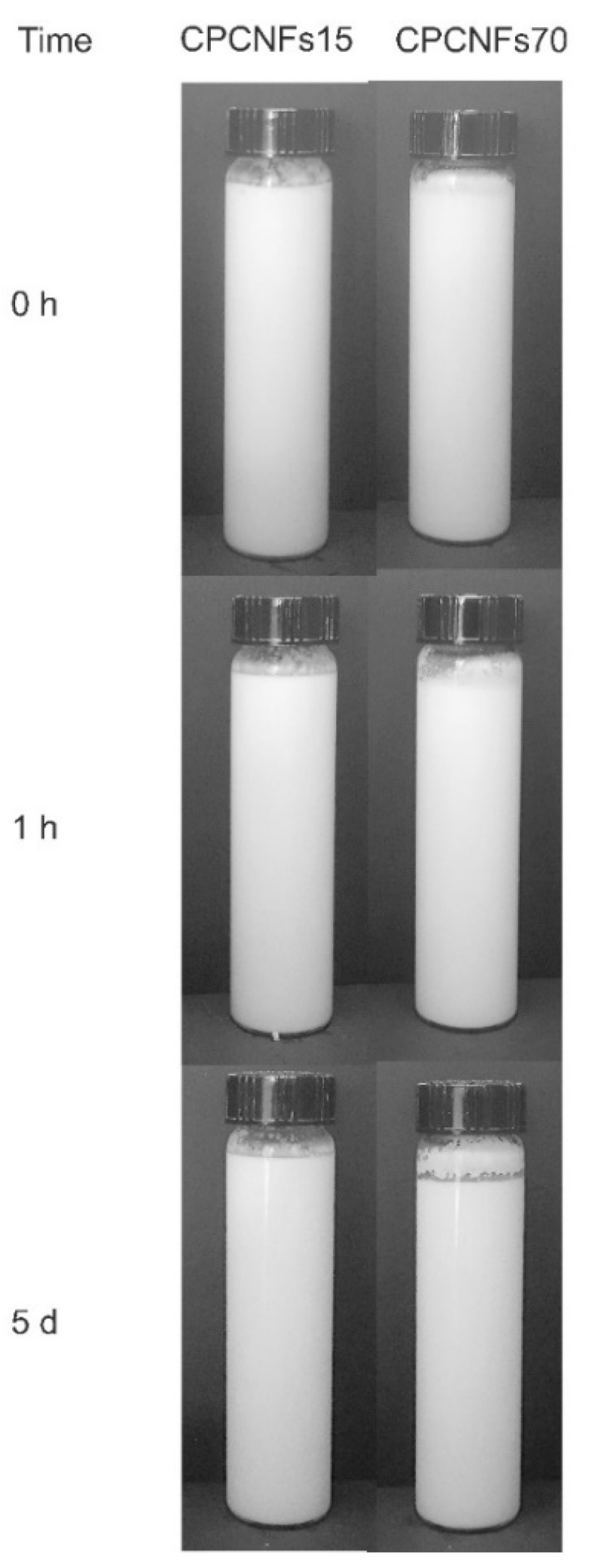
Emulsions processed with coconut oil and 0.6 wt.% of polysorbate 80 with 0.15 (CPCNFs15) and 0.7 wt.% (CPCNFs70) of CNFs, after 0 h, 1 h and 5 days of static storage.

The emulsions were prepared as described in Section 2.5, with a polysorbate concentration of 0.6 wt.%. The highest CNFs content resulted in a thick product, which adheres to the walls of the recipient, resulting in a crust observed above the emulsion after 5 days. However, the emulsions maintained their stability through the observations, as they do not separate after 5 days, even when a concentration of CNFs of 0.15 wt.% was used (Figure 4). This change in the behavior of the suspensions was further studied by fluorescence microscopy, after 1 day of manufacturing the emulsions.

**Figure 5:**
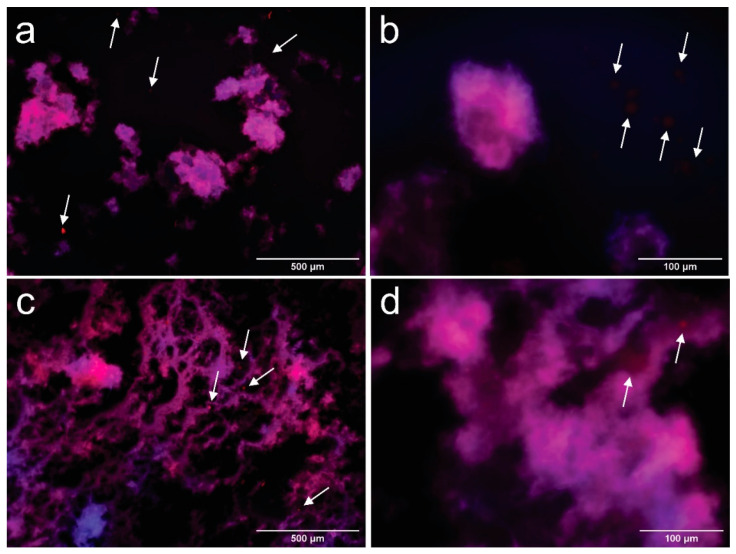
Fluorescence microscopy images of CPCNFs15 taken at 100× (**a**) and 400× (**b**) and CPCNFs70 taken at 100× (**c**) and 400× (**d**) after 1 day of their manufacture. CNFs are colored in blue and oil droplets are colored in red. Oil droplets outside the cellulosic structure are pointed by arrows in the images.

As observed in Figure 5, both samples show a dispersion of oil droplets, which are surrounded by the network of CNFs. In this case, the presence of a coalesced structure is not evident in either of the samples. However, the extent of the cellulosic network is increased in CPCNFs70 sample, which is coherent with the results obtained for samples without surfactant. Nevertheless, in this case oil structures spread through the cellulosic network (Figure 5c,d). A fraction of the droplets is freed to the surrounding medium, marked by arrows in Figure 5a,b. This phenomenon is clearer in CPCNFs15, as it has a lower amount of cellulose to form an entangled structure. This behavior indicates a fast adsorption of the surfactant over oil droplets [8], and their entrapment by the cellulosic network. This is further underscored by the presence of small and free oil droplets, which are absent in surfactant-free samples.

These results are coherent with previous works evaluating the role of surfactants in the stability of Pickering emulsions. Nesterenko et al. [2] evaluated the effect of Span™ 80 in the stabilization of paraffin oil in water emulsions with silica nanoparticles. They observed that higher contents of surfactant control the interfacial interactions, indicating their preferential adsorption over the oil surface. While Pichot et al. [3,8] noted that the presence of surfactants had an important effect in the microstructure of the emulsion, as they preferentially adsorb over the oil phase, controlling their further aggregation.

The use of ionic surfactants with CNFs and CNCs have been dominated by the electrostatic interactions between the charged groups present over the cellulosic structure, the surfactant of the hydrophilic group in the surfactant and the presence of counterions [23,27]. There is an important body of work related to the use of ionic surfactants to incorporate cellulose nanomaterials in hydrophobic matrices [27]. The presence of charged cellulose structures have been related to a decrease in the critical aggregation concentration (CAC) of the surfactants, due to the association between the alkane chain of the surfactant and the partially hydrophobic character of cellulose [23,46]. The use of cationic surfactants with CNC have shown a synergistic effect at low surfactant concentrations, as they cover the cellulosic surface, providing a more hydrophobic structure [7].

In the case of non-ionic surfactants, CNFs have been reported to weakly associate with the ethoxylated groups of non-ionic surfactants such as Triton™ X-100, forming bridges between micelles [46]. In the present case, polysorbate 80 avoided the coalescence of oil droplets during the mechanical processing of the emulsions, resulting in a finer dispersion.

**Figure 6:**
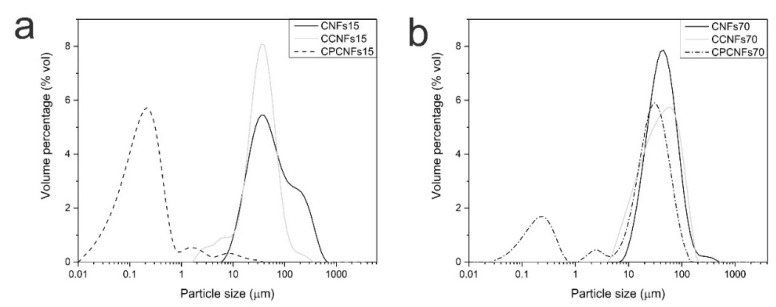
Particle size distribution of suspensions containing 0.15 wt.% (**a**) and 0.7 wt.% (**b**) of CNFs, with and without oil and surfactant.

In the absence of polysorbate 80, the emulsions were only stable after passing through the high-pressure homogenizer, while the addition of the surfactant allowed an emulsification after the Ultraturrax, indicating an improvement in emulsion stability when the amphiphilic compound was added, a similar behavior has been reported with sodium dodecyl sulfate as emulsifier [23]. The effect of surfactant in the stability and morphology of the emulsions in absence and with polysorbate 80 was assessed by laser diffraction as well, Figure 6a,b.

Before analyzing the emulsions, the distribution of CNFs without oil was assessed. CNFs suspensions exhibit a peak between 10 and 1000 µm, which is associated to the presence of cellulosic flocs [15], as mentioned in a previous section. This technique does not evaluate the diameter of the nanofibril, but focuses on the hydrodynamic diameter of the particle, therefore it serves as a comparison between the samples. These results are similar to previous reports on laser diffraction of CNFs from banana rachis [37,47,48].

When CNFs were mixed with coconut oil, particle size distribution shifts to lower diameters, with 1 vol.% of the particles below 10 μm, this change is related to minor quantities of oil outside the cellulosic network. These particles were observed by fluorescence microscopy (Figure 3). Some of these oil droplets are rather large, as the adsorption of CNFs to their surface is slower than small molecule surfactants, leading to droplet-droplet collisions and recoalescence during homogenization [15]. The introduction of a surfactant dramatically increases the percentage of droplets below 1 µm, indicating that it effectively stabilized the oil phase during emulsion processing, while some of these particles remain outside the cellulosic network [41].

**Figure 7:**
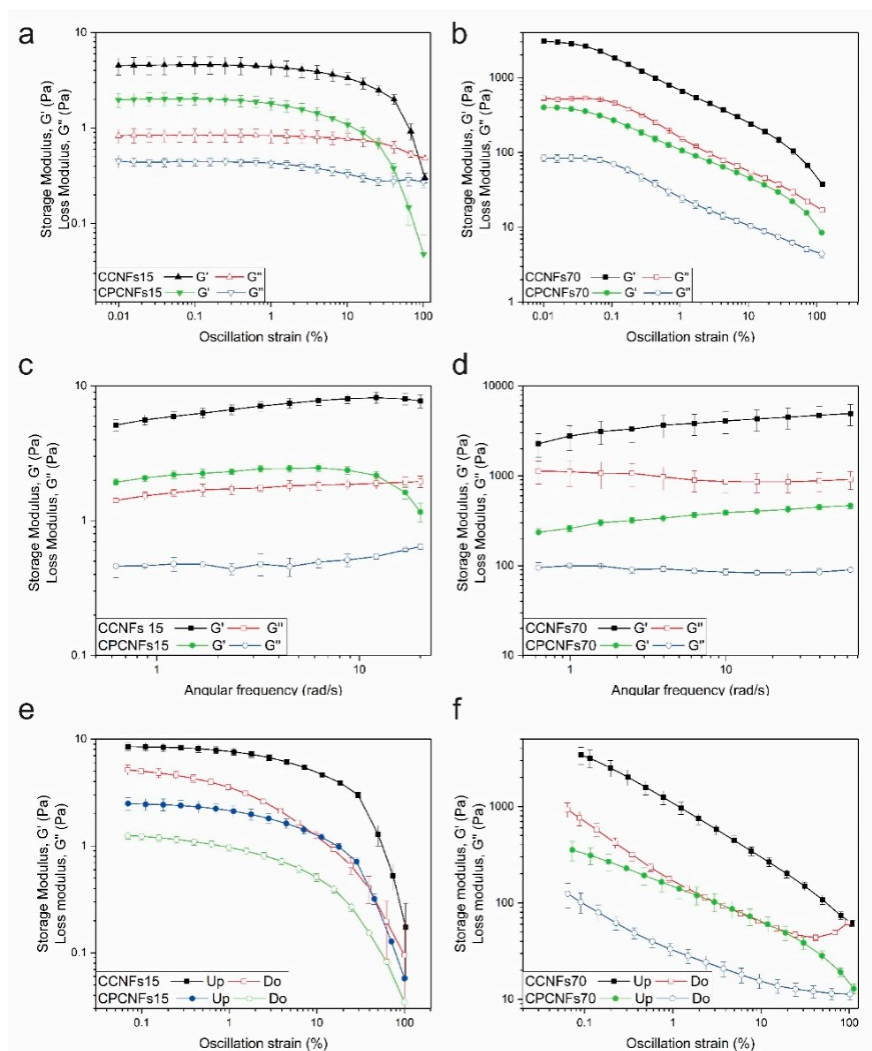
Rheological characterization of coconut oil emulsions with and without polysorbate 80 in the presence of 0.15 wt.% of CNFs, by amplitude (**a**) and frequency (**c**) sweeps and amplitude hysteresis (**e**) and in presence of 0.7 wt.% of CNFs, by amplitude (**b**) and frequency (**d**) sweeps and amplitude hysteresis (**f**). Hysteresis measurements taken with an upward amplitude are named as “Up” and with a downward amplitude are named as “Do”.

Particle size distribution is coherent with fluorescence microscopy of the suspensions with the surfactant. It shows that small droplets are formed and trapped inside the cellulosic network, higher quantities of CNFs can trap the dispersed phase more efficiently, explaining a smaller peak height at 0.2 µm in CPCNFs70, Figure 6b, compared to CPCNFs15, Figure 6a. Cellulose-non-ionic surfactant interactions has been reported as attractive in nature, as the cellulosic structure might adsorb the hydrophilic head of the surfactant [27], promoting the entrapment of oil in the cellulosic structure. It is coupled with a more entangled and gel-like network at a CNFs content of 0.7 wt.%. Rheological parameters of the emulsions with and without surfactant are observed in Figure 7a–f.

Due to possible problems with the creaming of CCNFs15, this sample and CPCNFs15 were evaluated using a vane and cup geometry. The samples tested form a gel-like network, Figure 7a,b. The extent of this network increases with the quantity of CNFs added, thus, G′ values are lower for suspensions with 0.15 wt.% of CNFs than for 0.7 wt.% ones. The lineal viscoelastic region (LVER) appears to be narrower for CCNFs70 and CPCNFs70. It forms a more entangled network that, coupled to the flow restriction imposed by oil droplets to the continuous phase; it leads to a more rigid and stiffer network with a decreased deformation [22,49]. After reaching the non-lineal zone, CCNFs15 and CPCNFs15 show a sharp decrease in G′, reaching a liquid-like consistency. CCNFs70 and CPCNFs70 showed two yield points. The first is associated to the breakage of interparticle bonding, resulting in the cleavage of the cellulosic network and the formation of clusters or flocs [50]. On the other hand, further deformation of the sample, and subsequent rupture of intercluster interactions explain the second yield point [50].

The formation of a stronger network is underscored by the frequency sweep. Initially, CCNFs15 and CPCNFs15 (Figure 7c) exhibit values of G′ above G″ up to 10 rad s^−1^, which is consistent with gel-like behavior [22]. After this frequency, G′ decreases since the gel network fractures. Due to poor reproducibility, frequency values above 20 rad s^−1^ were not evaluated for these samples. CCNFs70 and CPCNFs70 were studied at frequencies between 0.63 and 62.9 rad·s^−1^ and an amplitude of 0.02%, showing a gel-like behavior through the entire range (Figure 7b). A crossover point was not measured in the frequency sweep evaluated, evidencing that the structure was not largely broken by the analysis [22].

The increase of the elastic modulus with higher contents of CNFs is coherent with previous reports on the rheology of Pickering emulsions using nanocellulose [15,37]. Winnuprasith et al. [15] evaluated the addition of CNFs from mangosteen rind to 10 wt.% soybean oil in water emulsions. They reported an increase in G′ values with higher contents of CNFs, this modulus augmented almost three orders of magnitude when the cellulosic component varied from 0.05 to 0.7 wt.%. All the samples showed a gel-like behavior. The elastic character of the cellulosic suspensions was also studied by assessing the shear thixotropy of the emulsions. The thixotropy degree increased from 8.92 ± 3.18 at 0.05 wt.% to 808.45 ± 28.49 at 0.7 wt.%. This degree depends on the formation of a three-dimensional network which is broken down during the upward essay, and cannot reform completely during the downward measurement, firmer and more complex gels, require higher times to recover [15]. Thixotropy was reported as well for cellulose nanocrystals and bacterial cellulose suspensions, and it was associated to the breakage of the cellulosic network [41].

Another study added CNFs to *Curcuma longa* suspensions reported similar results, with an increase in the elastic behavior of the suspension. Increasing the concentration from 0.1 wt.% of CNFs to 0.9 wt.%, enhanced G′ by nearly 3 orders of magnitude and improved the stability of the resultant emulsion [37]. 

In the present work, the hysteresis of the resultant suspensions was also evaluated. After deforming the structure to an amplitude of 100%, and breaking the fibril network, it is observed that there is a recovery in G′ and G″ in all the samples evaluated (Figure 7e,f). Structural recovery is more marked in CCNFs15 and CPCNFs15, indicating a simpler aggregation (Figure 7e). CCNFs70 and CPCNFs70 show a lower recovery after breaking the structure, which is coherent with the complex structure mentioned in the amplitude sweep.

The addition of surfactant decreases G′ and G″ values in all the samples, which is associated with the surfactant adsorption over oil droplets, as it controls the cellulose–oil interactions [7]. The formation of smaller oil droplets and their dispersion within the CNFs network changes the structure of the emulsion, leading to a more hydrodynamic configuration and a lower resistance to deformation. A similar behavior has been reported in bentonite structures with CTAB [6]. High amounts of surfactant have been reported to affect the bridging of CNFs, reducing gel strength [13].

## 4. Conclusions

The present work explored the addition of a non-ionic surfactant in coconut oil emulsions with CNFs from banana rachis as Pickering emulsifier. Coconut oil emulsions in a buffer solution were prepared using different amounts of CNFs. Their morphological features were assessed, showing the formation of largely coalesced structures at nanocellulose contents up to 0.3 wt.%, creaming at these concentrations. A more elastic and rigid network was formed at higher amounts of cellulose. 

CNFs concentration affected the rheological behavior of the emulsions, forming a more rigid network and increasing the values of the storage (G′) and loss (G″) modulus with higher CNFs contents. The addition of polysorbate 80 as a non-ionic surfactant increased the stability and decreased the measured particle size of the emulsions. The surfactant readily adsorbs over the surface of oil during their breakage, stabilizing smaller droplets that are entrapped in the cellulosic network. The extent of entrapment depends on CNFs concentration, however, they do not appear to have an important role in emulsion stability through the duration of the study, as surfactants played a dominant role.

Rheological behavior of the obtained emulsions was affected by CNFs concentration and the presence of a non-ionic emulsifier, G′ and G″ values decreased for the suspensions obtained with 0.15 and 0.7 wt.% of CNFs. The incorporation of a polysorbate 80 can decrease the firmness of this network as observed in rheological measurements and controls droplet morphology, interfering with oil-cellulose interactions and modifying the physical properties of the resultant emulsion. This shall be considered during the formulation and processing of a commercial product.

## Data Availability

The data presented in this study are available on request from the corresponding author.

## Supplementary Materials

The following are available online at https://www.mdpi.com/article/10.3390/polym13213625/s1, Figure S1: Chemical structure of polysorbate 80. x + y + z + n = 20, Figure S2: Atomic force microscopy height image of dried CNFs (**a**), and a fiber diameter frequency histogram (**b**), Figure S3: Amplitude sweep of cellulose suspensions containing 0.15, 0.3, 0.45 and 0.7 wt.% of CNFs, after their homogenization, Table S1: Lipid profile of the coconut oil incorporated in the emulsions, Table S2: Surface tension of the coconut oil-buffer interphase, measured at various times and using different concentrations of polysorbate 80 dissolved in the aqueous phase.
