# Supplementary material for: Influence of a Non-Ionic Surfactant in the Microstructure and Rheology of a Pickering Emulsion Stabilized by Cellulose Nanofibrils
Source: Polymers (Basel). 2021 Oct 21;13(21):3625. doi: 10.3390/polym13213625 (PMC8587742; doi:10.3390/polym13213625)
Supplement: Supplementary file 1 [file polymers-13-03625-s001.zip › polymers-1407176-supplementary.pdf]

Supporting Information

Influence of a Non-Ionic Surfactant in the Microstructure and Rheology of a Pickering Emulsion Stabilized by Cellulose Nanofibrils

Jorge Velásquez-Cock [1,*], Angélica María Serpa [2], Catalina Gómez-Hoyos [1], Piedad Gañán [3], Manuel Romero-Sáez [4], Lina María Vélez [2], Natalia Correa-Hincapié [4] and Robin Zuluaga [2]

[1] Programa de Ingeniería en Nanotecnología, Universidad Pontificia Bolivariana, Medellín 050031, Colombia; catalina.gomezh@upb.edu.co
[2] Facultad de Ingeniería Agroindustrial, Universidad Pontificia Bolivariana, Medellín 050031, Colombia; angelicaserpa31@gmail.com (A.M.S.); lina.velez@upb.edu.co (L.M.V.); robin.zuluaga@upb.edu.co (R.Z.)
[3] Facultad de Ingeniería Química, Universidad Pontificia Bolivariana, Medellín 050031, Colombia; piedad.ganan@upb.edu.co
[4] Grupo Calidad, Metrología y Producción, Instituto Tecnológico Metropolitano, Medellín 050034, Colombia; manuelromero@itm.edu.co (M.R.-S.); nataliacorrea@itm.edu.co (N.C.-H.)
* Correspondence: jorgeandres.velasquez@upb.edu.co

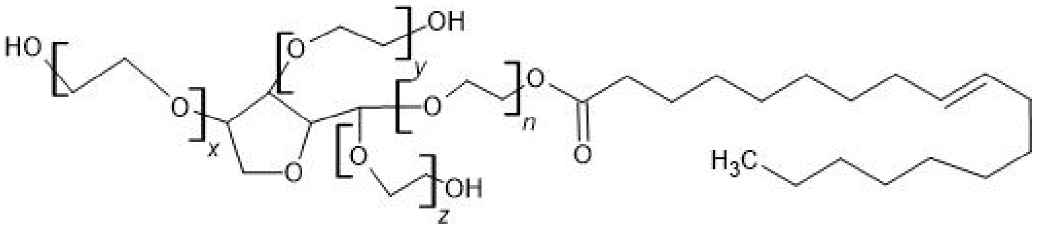

Figure S1. Chemical structure of polysorbate 80. x + y + z + n = 20.

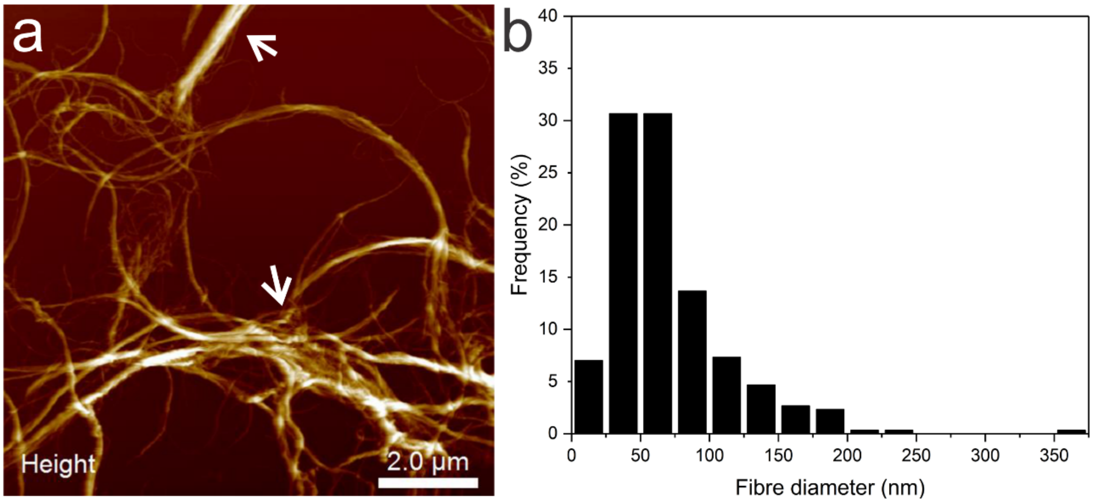

Figure S2. Atomic force microscopy height image of dried CNFs (**a**), and a fiber diameter frequency histogram (**b**).

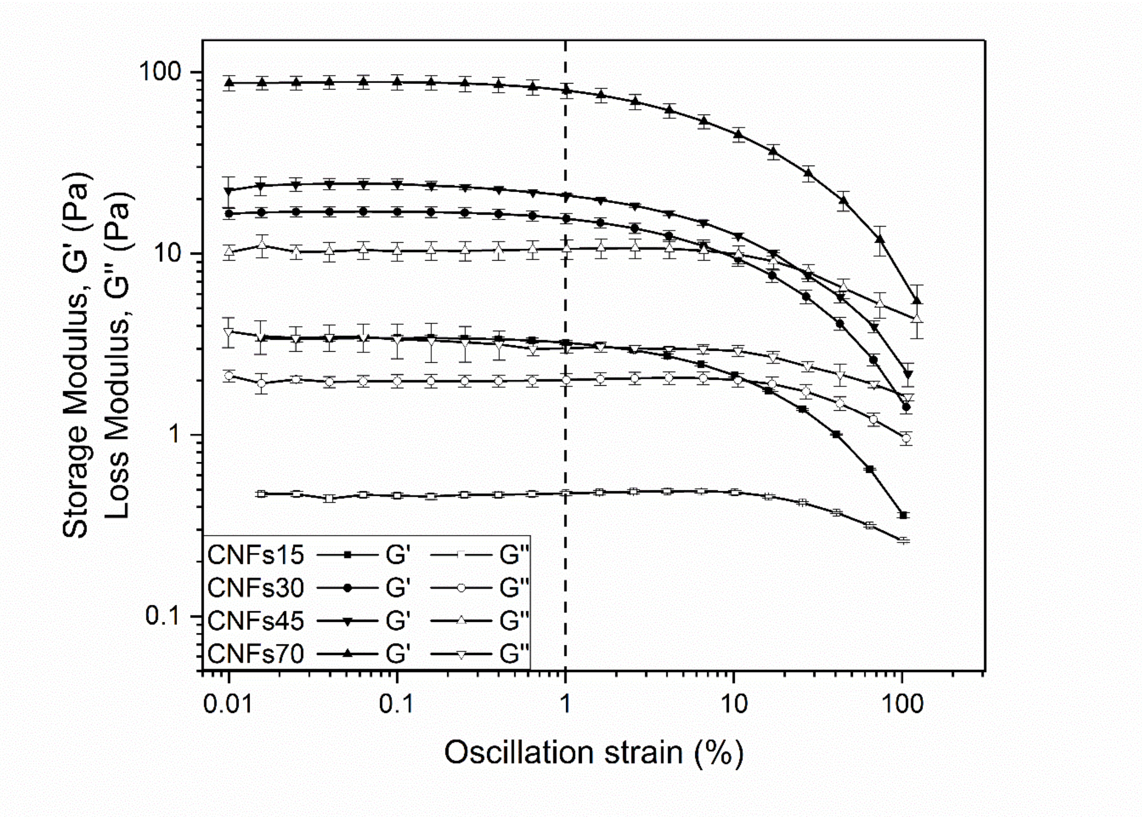

Figure S3. Amplitude sweep of cellulose suspensions containing 0.15, 0.3, 0.45 and 0.7 wt.% of CNFs, after their homogenization.

Table S1. Lipid profile of the coconut oil incorporated in the emulsions.

Fatty Acid	Fatty Acid Content (g/100 g)
C6:0	0.535 ± 0.002
C8:0	7.058 ± 0.017
C10:0	5.855 ± 0.017
C12:0	49.145 ± 0.022
C14:0	17.523 ± 0.024
C16:0	7.652 ± 0.025
C17:0	Not detected
C18:0	2.851 ± 0.004
C20:0	Not detected
C16:1	Not detected
C18:1n9c	4.310 ± 0.009
C20:1n9	Not detected
C18:2n6c	0.692 ± 0.002
C18:3n3	Not detected

Table S2. Surface tension of the coconut oil-buffer interphase, measured at various times and using different concentrations of polysorbate 80 dissolved in the aqueous phase.

Time (min)	Surface Tension (dyn cm^{-1})		
	**0**	**15**	**30**
**0 wt.%**	15.81 ± 2.04	5.84 ± 0.74	4.12 ± 0.49
**0.1 wt.%**	2.94 ± 0.77	0.81 ± 0.13	0.61 ± 0.05
**0.2 wt.%**	3.09 ± 0.45	0.85 ± 0.08	0.63 ± 0.07
**0.4 wt.%**	2.47 ± 0.13	0.72 ± 0.04	0.7 ± 0.06
**0.6 wt.%**	2.19 ± 0.13	0.55 ± 0.02	0.52 ± 0.02
**1 wt.%**	1.77 ± 0.08	0.57 ± 0.01	0.55 ± 0.01
